# Obesity and dysglycemia independently predict symptom burden but not satisfaction with care in polycystic ovary syndrome: a cross-sectional study

**DOI:** 10.1007/s00404-025-08219-9

**Published:** 2025-10-17

**Authors:** Annette Bachmann, Julia Estermann, Marina Sourouni, Thomas Karn, Norman Bitterlich, Susanna Weidlinger, Petra Stute

**Affiliations:** 1 Division of Reproductive Endocrinology, Department of Gynecology and Obstetrics, University Hospital Frankfurt, Goethe University, Frankfurt, Germany; 2 Faculty of Medicine, University of Bern, Bern, Switzerland; 3 Department for Gynaecological Endocrinology and Fertility Disorders, University Hospital Heidelberg, Heidelberg, Germany; 4 Freelance statistician ‘Statistics – analysis, Consulting, Training’, Chemnitz, Germany; 5 Division of Gynaecological Endocrinology and Reproductive Medicine, University Hospital Inselspital, Bern, Switzerland; 6 Present Address: Department of Anesthesiology and Intensive Care, Lucerne Cantonal Hospital, Lucerne, Switzerland

**Keywords:** PCOS, Obesity, Body mass index, Dysglycemia, Patient satisfaction, Counselling gaps

## Abstract

**Purpose:**

Polycystic ovary syndrome (PCOS) is associated with a broad range of reproductive, metabolic, and psychosocial symptoms. This study aimed to evaluate whether body mass index (BMI) and dysglycemia independently predict symptom burden and to explore how these factors relate to patient-perceived care gaps, as reflected by satisfaction with counselling.

**Methods:**

We conducted a cross-sectional analysis of 1,926 self-identified women with PCOS. Participants reported symptom prevalence and rated satisfaction with counselling across six clinical domains using a 0–100 scale. Logistic regression models assessed associations between BMI, dysglycemia (prediabetes, gestational diabetes, type 2 diabetes), and PCOS symptoms. Multivariate linear regression was used to identify predictors of satisfaction with counselling.

**Results:**

The most frequently reported symptoms were body image dissatisfaction (78.2%), hirsutism (75.0%), and menstrual irregularity (65.0%). Both higher BMI and dysglycemia were independently associated with increased odds of infertility (BMI: OR 1.48, 95% CI 1.38–1.58, *p* < 0.001; dysglycemia: OR 1.85, 95% CI 1.50–2.28, *p* < 0.001), as well as with depression, anxiety, alopecia, and hirsutism (all *p* < 0.001)Dysglycemia was not significantly associated with acne or menstrual irregularity. Mean satisfaction with PCOS counselling was low (35.1 ± 26.9), with the lowest scores reported for aesthetic concerns (31.4 ± 27.8). In adjusted models, depression, alopecia, body image dissatisfaction, and hirsutism were independently associated with lower satisfaction. BMI and dysglycemia were not independently associated with satisfaction after accounting for symptoms.

**Conclusion:**

While obesity and dysglycemia contribute to increased PCOS symptom burden, they do not explain low satisfaction with care. Perceived care gaps are most pronounced in emotional and aesthetic domains, indicating the need for more holistic, patient-centered PCOS care models.

**Supplementary Information:**

The online version contains supplementary material available at 10.1007/s00404-025-08219-9.

## What does this study add to the clinical work?

**Table:** 

BMI and dysglycemia are independent predictors of PCOS symptom burden, but do not account for patients’ primary sources of distress. Instead, cosmetic and mental health symptoms are more closely linked to dissatisfaction with care. These findings call for a shift toward truly patient-centered, interdisciplinary PCOS management, with explicit attention to emotional and aesthetic concerns that are often overlooked in routine practice.	

## Introduction

Polycystic ovary syndrome (PCOS) is a common endocrine disorder affecting 10–13% of women of reproductive age, characterized by hyperandrogenism, ovulatory dysfunction, and polycystic ovarian morphology on ultrasound [1]. Beyond its reproductive manifestations, PCOS is increasingly recognized as a complex metabolic condition. A substantial proportion of affected women exhibit dysglycemia independent of body mass index (BMI) [2, 3]. Obesity is also highly prevalent, with rates ranging from 38 to 88% depending on geographic, ethnic, and diagnostic context [4]. Excess weight worsens both reproductive and metabolic phenotypes and is associated with higher rates of infertility, cardiovascular risk, and psychological distress [5–7]. Importantly, obesity can impair fertility even in the absence of PCOS [8, 9]. Compared with women without PCOS, those affected are more likely to develop central adiposity, experience progressive weight gain, and exhibit insulin resistance [6, 7]. Central fat accumulation, particularly increased abdominal fat mass, has further been linked to more severe hyperandrogenism in PCOS [10].

In addition to obesity-related risk, PCOS is intrinsically associated with impaired glucose metabolism, including impaired fasting glucose, impaired glucose tolerance, and type 2 diabetes mellitus (T2DM). Cohort studies report that 12–13% of women with PCOS have prediabetes and 1.5–10% develop T2DM-rates significantly higher than in age-matched controls [3, 11]. These risks are not confined to overweight or obese phenotypes, underscoring an inherent predisposition to metabolic dysfunction [2].

Despite the documented disease burden, substantial gaps persist in clinical management and patient-perceived support. The 2018 international guideline for the assessment and management of PCOS, updated in 2023, emphasizes individualized, multidisciplinary care that addresses metabolic health, emotional well-being, and aesthetic concerns [1]. However, patient reports indicate that current care often falls short of these recommendations, particularly regarding lifestyle counseling, mental health, and cosmetic management [12, 13]. To address these issues, we conducted a cross-sectional survey of women with PCOS to evaluate perceived disease burden and quality of care. The present analysis had two primary aims: (A) to assess whether BMI and dysglycemia (defined as either prediabetes, gestational diabetes, or type 2 diabetes) are independent predictors of common PCOS symptoms, and (B) to identify which symptoms are perceived as most burdensome by patients, using satisfaction with care as a proxy for unmet clinical needs.

## Materials and methods

This cross-sectional cohort study was conducted via an online survey between January 1 and March 14, 2021, in German speaking Europe. The survey link was disseminated through PCOS-related self-help forums and support groups on social media, as well as university newsletters and hospital websites. A study flyer and the survey web link are available in the supplementary materials. Eligible participants were women aged 18 years or older, fluent in German, and either previously diagnosed with PCOS by a physician or meeting the revised Rotterdam ESHRE/ASRM consensus diagnostic criteria for PCOS. Women were excluded if they were postmenopausal or had a known diagnosis of other causes of hyperandrogenism.

### Questionnaire development

The questionnaire (Supplementary File 1) was developed through multiple iterative steps, as previously described by our group [14]. Initial item generation was guided by the domains outlined in the 2018 international PCOS guideline. The preliminary version was reviewed and pilot-tested by 10 volunteers, with revisions made based on their feedback. It was subsequently evaluated by a statistician and finalized through consensus among all co-authors.

The questionnaire comprised eight domains according to recommendations of the international guideline (1) demographics, (2) diagnostic criteria, (3) aesthetic concerns, (4) metabolic health, (5) menstrual cycle characteristics, (6) reproductive health, (7) mental health, and (8) prevention and monitoring of long-term risks such as endometrial cancer and elevated cardiovascular risk. Each domain included follow-up questions assessing symptom burden, number of medical consultations, prior treatments, and perceived quality of care.

To minimize bias, all questions were neutrally phrased and written in lay-accessible language. Participants were blinded to the study hypothesis. Input validation was used to ensure data quality, and response options included “unknown” where appropriate. The questionnaire was implemented using REDCap software to ensure secure and standardized data collection.

### Assessment criteria

In this analysis body image concerns were assessed using two established screening questions for body dysmorphic disorder (BDD): “Do you worry a lot about the way you look and wish you could think about it less?” and “On a typical day, do you spend more than one hour worrying about your appearance?” [15] A positive response to either item was considered indicative of body image dissatisfaction.

Aesthetic symptoms were assessed by asking participants to indicate whether they currently or previously experienced any of the following cosmetic concerns: excessive body hair (hirsutism), hair loss (alopecia), or acne. A positive response to any item was recorded as the presence of the respective symptoms.

Depressive symptoms were assessed using two items adapted from the Patient Health Questionnaire-2 (PHQ-2), evaluating loss of interest and feelings of hopelessness over the past two weeks. A response score of ≥ 3 on either item (indicating symptoms present on at least half of the days) was considered indicative of depression.

Anxiety symptoms were assessed using two items from the *Generalized Anxiety Disorder Scale (GAD-7)*—“feeling nervous, anxious, or on edge” and “not being able to stop or control worrying”—with participants rating frequency on a 5-point Likert scale from 1 (never) to 5 (constantly/almost every day) [16].

Menstrual irregularity was assessed by asking whether the participant’s cycle length was consistently the same (± 7 days); a “no” response was considered indicative of irregularity.

Infertility was defined as a self-reported history of attempting to conceive for more than one year without success, based on the question: “Have you ever tried to become pregnant for over one year without success?” A “yes” response was considered indicative of infertility.

Dysglycemia was defined as the self-reported presence of elevated fasting blood glucose (prediabetes), gestational diabetes, or type 2 diabetes in accordance with the American Diabetes Association (ADA) definitions. Elevated fasting glucose and a history of gestational diabetes were classified as risk factors for type 2 diabetes [17].

### Statistical analysis

An a priori power analysis determined that a minimum sample of 199 participants would be required to detect a small effect size (Cohen's d = 0.1) with 80% power. Data collection continued until survey response rates declined. Descriptive statistics were used to characterize the cohort. Categorical variables were compared using Chi-square tests. Continuous and ordinal variables were analyzed with Mann–Whitney U tests due to non-normal distributions. Binary logistic regression models were applied to assess independent associations between BMI, dysglycemia, and the presence of selected PCOS symptoms (e.g., infertility, hirsutism, alopecia, depression). Satisfaction scores (0–100 scale) were treated as continuous variables and used as outcome for linear regression. Spearman’s rho was used to examine correlations between BMI categories and satisfaction scores across care domains. All statistical tests were performed using a significance level of *p* < 0.05. Owing to the exploratory nature of the study, corrections for multiple testing were not applied. As not all participants responded to every question, the number of responses (*n*) is reported for each analysis. Missing data was addressed using listwise deletion. Data analyses were conducted using SPSS, version 27.0 (IBM Corp., Armonk, NY, USA).

## Results

### Cohort characteristics

A total of 1942 of 2029 participants with self-reported PCOS answered all questions on dysglycemia. We excluded 16 cases with Type I diabetes or inconsistent information on dysglycemia leading to a final cohort of 1926 participants (Supplementary Fig. S1). The mean age was 28.9 years (SD 5.5), and the mean BMI was 30.5 kg/m^2^ (SD 8.5). One-third of participants (33.3%) reported dysglycemia, including prediabetes (24.1%), gestational diabetes (6.8%), and type 2 diabetes (2.4%) (Table 1).

**Table 1:** Characteristics of the cohort

Parameter		*N* = 1926 (%)
Age	Mean [SD] years	28.9 [5.5]
BMI^a^	Mean [SD] kg/m^2^	30.5 [8.5]
BMI classification	< 25	639 (33.2%)
	25–29	347 (18.0%)
	30–34	372 (19.3%)
	≥ 40	276 (14.3%)
	Adip-3	291 (15.1%)
Smoking	No	1545 (80.2%)
	Yes	381 (19.8%)
Domicile	Switzerland	384 (19.9%)
	Germany	1395 (72.4%)
	Austria	123 (6.4%)
	Other	24 (1.2%)
Ethnicity	White (only or in combination)	1785 (92.7%)
	Other (hispanic, arabic, asian, black)	141 (7.3%)
Education	Secondary level 1, obligatory	447 (23.2%)
	Secondary level 2, universal education	326 (16.9%)
	Secondary level 2, vocational education	292 (15.2%)
	Tertiary level, high vocational education	180 (9.3%)
	Tertiary level, university	680 (35.3%)
	Other	1 (0.1%)
Employment status	Employed	1732 (89.9%)
	Unemployed	194 (10.1%)
Partnership status	In a partnership	769 (39.9%)
	Not in a partnership	1157 (60.1%)
Parity	No	1356 (70.4%)
	Yes	570 (29.6%)
Infertility	No	1057 (56.0%)
	Yes	832 (44.0%)
Menstrual irregularity	No	482 (35.0%)
	Yes	894 (65.0%)
Acne	No	701 (36.4%)
	Yes	1225 (63.6%)
Alopecia	No	1123 (58.3%)
	Yes	803 (41.7%)
Hirsutism	No	482 (25.0%)
	Yes	1444 (75.0%)
Depression	No	726 (37.7%)
	Yes	1200 (62.3%)
Anxiety	No	678 (35.2%)
	Yes	1248 (64.8%)
Body image	No	420 (21.8%)
	Yes	1506 (78.2%)
Dysglycemia	No	1284 (66.7%)
	Yes	642 (33.3%)
Dysglycemia type	Type 2 diabetes	46 (2.4%)
	Gestational diabetes	131 (6.8%)
	Prediabetes	465 (24.1%)
	None	1284 (66.7%)

^a^Body mass index (kg/m^2^)

The most frequently reported concerns among women with PCOS in this cohort were dissatisfaction with body image (78.2%), hirsutism (75.0%), and menstrual irregularity (65.0%). (Supplementary Fig. S2).

### Associations of PCOS symptoms with obesity and dysglycemia

**Fig. 1:**
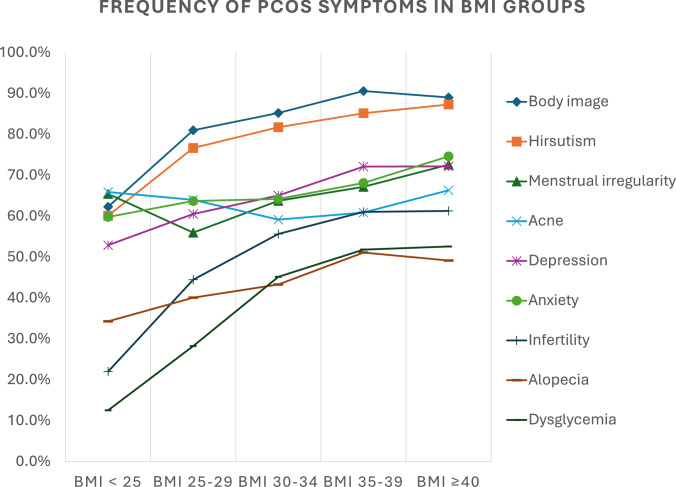
Frequency of PCOS symptoms stratified by BMI. Prevalence of self-reported symptoms among participants with polycystic ovary syndrome (PCOS) stratified by body mass index (BMI) groups: < 25, 25–29, 30–34, 35–39, and ≥ 40 kg/m^2^. Symptoms assessed included body image concerns, hirsutism, menstrual irregularity, acne, depression, anxiety, infertility, alopecia, and dysglycemia. Higher BMI was generally associated with increased prevalence of body image concerns, hirsutism, depression, and dysglycemia, while acne prevalence showed less variation across BMI groups

As shown in Table 2 and Fig. 1, the prevalence of nearly all PCOS symptoms increased significantly across BMI categories, with the strongest gradients observed for infertility (22.0% in BMI < 25 vs. 61.3% in BMI ≥ 40) and dysglycemia (12.5% vs. 52.6%, respectively). In contrast, acne showed no significant association with BMI (*p* = 0.166).

**Table 2:** Association of body mass index and dysglycemia with main symptoms of PCOS

Main PCOS symptoms		BMI^a^ < 25	BMI 25–29	BMI 30–34	BMI 35–39	BMI ≥ 40	*P*-value*
Body image	No	241 (37.7%)	66 (19.0%)	55 (14.8%)	26 (9.4%)	32 (11.0%)	** < 0.001**
	Yes	398 (62.3%)	281 (81.0%)	317 (85.2%)	250 (90.6%)	259 (89.0%)	
Hirsutism	No	255 (39.9%)	81 (23.3%)	68 (18.3%)	41 (14.9%)	37 (12.7%)	** < 0.001**
	Yes	384 (60.1%)	266 (76.7%)	304 (81.7%)	235 (85.1%)	254 (87.3%)	
Menstrual irregularity	No	140 (34.6%)	107 (44.0%)	99 (36.3%)	68 (32.9%)	68 (27.4%)	**0.004**
	Yes	265 (65.4%)	136 (56.0%)	174 (63.7%)	139 (67.1%)	180 (72.6%)	
Acne	No	218 (34.1%)	125 (36.0%)	152 (40.9%)	108 (39.1%)	98 (33.7%)	0.166
	Yes	421 (65.9%)	222 (64.0%)	220 (59.1%)	168 (60.9%)	193 (66.3%)	
Depression	No	301 (47.1%)	137 (39.5%)	130 (34.9%)	77 (27.9%)	81 (27.8%)	** < 0.001**
	Yes	338 (52.9%)	210 (60.5%)	242 (65.1%)	199 (72.1%)	210 (72.2%)	
Anxeity	No	257 (40.2%)	126 (36.3%)	133 (35.8%)	88 (31.9%)	74 (25.4%)	** < 0.001**
	Yes	382 (59.8%)	221 (63.7%)	239 (64.2%)	188 (68.1%)	217 (74.6%)	
Infertility	No	489 (78.0%)	190 (55.6%)	161 (44.4%)	106 (39.0%)	110 (38.7%)	** < 0.001**
	Yes	138 (22.0%)	152 (44.4%)	202 (55.6%)	166 (61.0%)	174 (61.3%)	
Alopecia	No	420 (65.7%)	208 (59.9%)	211 (56.7%)	135 (48.9%)	148 (50.9%)	** < 0.001**
	Yes	219 (34.3%)	139 (40.1%)	161 (43.3%)	141 (51.1%)	143 (49.1%)	
Dysglycemia	No	559 (87.5%)	249 (71.8%)	204 (54.8%)	133 (48.2%)	138 (47.4%)	** < 0.001**
	Yes	80 (12.5%)	98 (28.2%)	168 (45.2%)	143 (51.8%)	153 (52.6%)	

^a^Body Mass index ( BMI) in kg/m^2^

*Bold indicates statistical significance

Dysglycemia was associated with higher prevalence of multiple PCOS symptoms. Compared to normoglycemic participants, those with dysglycemia (risk or confirmed type 2 diabetes) reported substantially higher rates of infertility (73.3% in type 2 diabetes vs. 36.1% in normoglycemia), alopecia (63.0% vs. 37.6%), depression (71.7% vs. 58.7%), and hirsutism (89.1% vs. 70.6%) (Supplementary Table S1 and Supplementary Fig. S3).

Logistic regression analyses confirmed that both BMI and dysglycemia were independent predictors of key PCOS symptoms. In bivariate models adjusting for each other, both BMI and dysglycemia remained significantly associated with infertility, alopecia, hirsutism, depression, anxiety, and body image dissatisfaction. BMI was entered as an ordinal variable (five categories), and odds ratios (ORs) represent the linear trend per category increase in BMI. For example, the OR for infertility was 1.48 (95% CI: 1.38–1.58; *p* < 0.001) per BMI category increase, while for dysglycemia the OR was 1.85 (95% CI: 1.50–2.28; *p* < 0.001). No independent associations were observed for acne or menstrual irregularity (Table 3).

**Table 3:** Logistic regression analysis of main PCOS symptoms by BMI and dysglycemia

PCOS symptom	Factor	OR	95% CI	*P*-value*	OR	95% CI	*P*-value*
		Univariate			Bivariate		
Infertility	BMI (5 groups)^a^	1.57	1.47–1.68	** < 0.001**	1.48	1.38–1.58	** < 0.001**
	Dysglycemia^b^	2.63	2.16–3.21	** < 0.001**	1.85	1.50–2.28	** < 0.001**
Alopecia	BMI (5 groups)	1.19	1.12–1.27	** < 0.001**	1.14	1.07–1.22	** < 0.001**
	Dysglycemia	1.65	1.36–2.00	** < 0.001**	1.44	1.17–1.76	** < 0.001**
Depression	BMI (5 groups)	1.26	1.18–1.34	** < 0.001**	1.22	1.14–1.31	** < 0.001**
	Dysglycemia	1.60	1.31–1.96	** < 0.001**	1.31	1.06–1.62	**0.014**
Anxiety	BMI (5 groups)	1.16	1.09–1.24	** < 0.001**	1.11	1.03–1.19	**0.004**
	Dysglycemia	1.68	1.37–2.07	** < 0.001**	1.51	1.22–1.88	** < 0.001**
Hirsutism	BMI (5 groups)	1.52	1.40–1.65	** < 0.001**	1.46	1.34–1.59	** < 0.001**
	Dysglycemia	2.13	1.67–2.71	** < 0.001**	1.47	1.14–1.91	**0.003**
Body image	BMI (5 groups)	1.63	1.49–1.78	** < 0.001**	1.58	1.43–1.73	** < 0.001**
	Dysglycemia	2.09	1.62–2.69	** < 0.001**	1.34	1.02–1.76	**0.037**
Menstrual irregularity	BMI (5 groups)	0.91	0.85–0.99	**0.020**	0.91	0.84–0.99	**0.028**
	Dysglycemia	0.92	0.73–1.16	0.464	1.01	0.79–1.29	0.970
Acne	BMI (5 groups)	0.98	0.92–1.04	0.466	1.00	0.93–1.07	0.938
	Dysglycemia	0.82	0.68–1.00	0.052	0.83	0.67–1.02	0.074

^a^Body Mass index (BMI) in kg/m^2^ stratified in 5 groups (< 25, 25–29, 30–34, 35–39, ≥ 40)

^b^Dysglycemia: elevated fasting glucose, gestational diabetes, or type 2 diabetes

*Bold indicates statistical significance

### Patient satisfaction and symptom burden

We next sought to determine which PCOS-related symptoms were perceived as most burdensome by patients. To address this question, we used patient satisfaction scores with counselling on various PCOS-related complaints—measured on a scale from 0 to 100—as a proxy for symptom-related burden.

Table 4 and Supplementary Fig. S4 present the mean satisfaction scores across different domains of counselling, reflecting the areas of greatest perceived support or unmet need. Overall satisfaction with PCOS-related counselling was low, with a mean score of 35.1 (SD 26.9) on a 100-point scale. The highest satisfaction score was for counselling on fertility (49.5, SD 30.7), while the lowest was for aesthetic aspects (31.4, SD 27.8). The individual satisfaction scores across different domains of counselling were highly correlated (Supplementary Table S2). In a multivariate linear regression model, satisfaction with counselling on PCOS associated risks including cardiovascular disease and endometrial cancer (*B* = 0.261, 95% CI: 0.203–0.320), glucose metabolism (*B* = 0.187), aesthetic aspects (*B* = 0.163), fertility (*B* = 0.162), and the menstrual cycle (*B* = 0.135) were all independently associated with overall satisfaction (all *p* < 0.001). Mental health counselling showed only a trend toward significance (*p* = 0.084) (Supplementary Table S3).

**Table 4:** Mean values of satisfaction scores

Satisfaction with counselling regarding	Mean	SD	N
Fertility	49.54	30.67	1889
Menstrual cycle	45.47	29.10	1913
Mental health	36.48	29.67	1856
Long-term risks	34.63	28.20	1819
Glucose metabolism	34.29	30.29	642
Aesthetic aspects	31.41	27.75	1926
Overall counselling regarding PCOS	35.12	26.86	1786

We analyzed correlations between satisfaction scores and BMI. As shown in Supplementary Table S4, higher BMI was significantly, though only weakly, associated with lower satisfaction across most counselling domains. The strongest negative correlations, albeit negligible in magnitude, were observed for mental health (ρ = 0.113, *p* < 0.001), fertility (ρ = 0.112, *p* < 0.001), and aesthetic aspects (ρ = 0.109, *p* < 0.001).

**Fig. 2:**
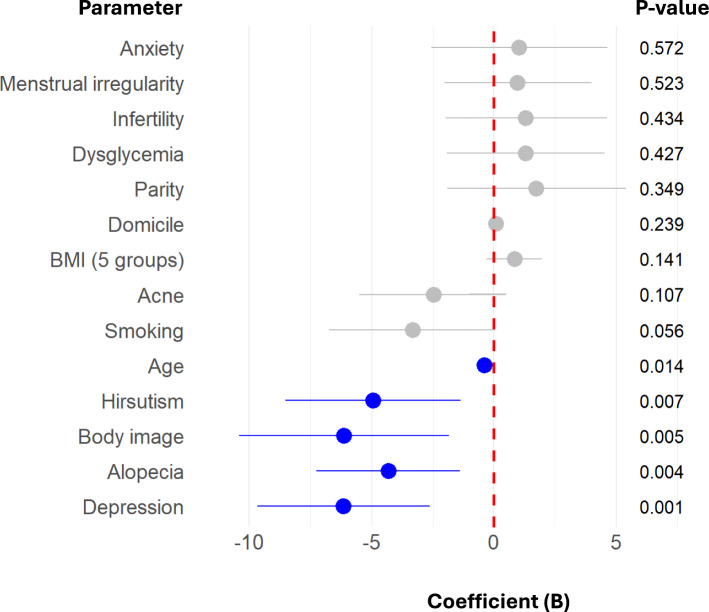
Multivariate regression analysis of overall satisfaction score. Forest plot showing coefficients (B) and 95% confidence intervals from multivariable regression analysis assessing associations between clinical, demographic, and lifestyle parameters and overall satisfaction score. Parameters with statistically significant associations (*p* < 0.05) are shown in blue, including age, hirsutism, body image concerns, alopecia, and depression. Non-significant parameters are shown in grey. The red dashed vertical line indicates no effect (*B* = 0)

In multivariate analysis of overall satisfaction (Table 5, Fig. 2), depression (*B* = 5.72, *p* < 0.001), body image dissatisfaction (*B* = 6.14, *p* = 0.005), alopecia (*B* = 4.32, *p* = 0.004), and hirsutism (*B* = 4.95, *p* = 0.007) independently predicted lower scores. In contrast, BMI and dysglycemia were not associated with overall satisfaction after accounting for symptom burden.

**Table 5:** Multivariate regression analysis of overall satisfaction score by baseline parameters and symptoms

(n = 1274)	Coefficient B	(95% CI)	*P*-value*
Depression	− 6.15	(− 9.68 … − 2.62)	**0.001**
Alopecia	− 4.32	(− 7.26 … − 1.38)	**0.004**
Body image	− 6.14	(− 10.42 … − 1.85)	**0.005**
Hirsutism	− 4.95	(− 8.52 … − 1.37)	**0.007**
Age	− 0.38	(− 0.69 … − 0.08)	**0.014**
Smoking	− 3.33	(− 6.75 … 0.08)	0.056
Acne	− 2.48	(− 5.50 … 0.53)	0.107
BMI (5 groups)^a^	0.85	(− 0.28 … 1.98)	0.141
Domicile	0.09	(− 0.06 … 0.24)	0.239
Parity	1.74	(− 1.91 … 5.40)	0.349
Dysglycemia^b^	1.31	(− 1.92 … 4.53)	0.427
Infertility	1.31	(− 1.98 … 4.61)	0.434
Menstrual irregularity	0.98	(− 2.03 … 4.00)	0.523
Anxiety	1.04	(− 2.56 … 4.63)	0.572

^a^Body Mass index (BMI) in kg/m^2^ stratified in 5 groups (< 25, 25–29, 30–34, 35–39, ≥ 40)

^b^Dysglycemia: elevated fasting glucose, gestational diabetes, or type 2 diabetes

*Bold indicates statistical significance

## Discussion

In this cross-sectional survey of women with PCOS, we found that both BMI and dysglycemia (defined as either prediabetes, gestational diabetes, or type 2 diabetes) were independently associated with an increased prevalence of key PCOS symptoms, including infertility, alopecia, hirsutism, depression, and body image dissatisfaction. Logistic regression confirmed that these associations persisted after mutual adjustment, suggesting that BMI and dysglycemia contribute independently to PCOS symptom burden rather than acting solely as correlated risk factors. The strongest associations were observed for infertility and hirsutism, with women in the highest BMI category nearly three times more likely to report infertility compared with those of normal weight. Dysglycemia was additionally linked to higher rates of alopecia and depressive symptoms. These findings reinforce prior evidence that metabolic dysfunction in PCOS is both common and clinically meaningful, even in lean phenotypes [2, 3]. However, BMI did not independently predict overall satisfaction with care in our cohort, despite weak but significant negative correlations with most satisfaction domains. This contrasts with a previously published fertility-focused subanalysis of 564 participants from the same dataset, where BMI was the sole predictor of dissatisfaction with fertility counselling [18]. The discrepancy likely reflects contextual differences and highlights the limitations of BMI as a surrogate for adiposity and metabolic risk. BMI does not distinguish between lean and fat mass or account for fat distribution, particularly visceral adiposity, which is highly relevant in PCOS. Prior studies have shown that women with PCOS may display insulin resistance and metabolic disturbances even at normal BMI levels, and that BMI correlates poorly with hyperandrogenism and phenotype severity [19–21].

In contrast to the metabolic emphasis often prioritized in clinical settings, our analysis of patient-reported outcomes revealed that psychosocial and aesthetic symptoms were stronger predictors of dissatisfaction with care. Hirsutism, alopecia, depression, and body image concerns emerged as the most burdensome symptoms, aligning with findings by Hofmann et al., who reported high rates of body image distress and poor mental health in women with PCOS, with substantial effects on quality of life [22]. Similarly, Estermann et al. reported high unmet needs and low satisfaction in patients experiencing aesthetic manifestations of PCOS, particularly regarding appearance and emotional support [14]. Our findings further support these observations by demonstrating that depression and body image dissatisfaction were among the strongest independent predictors of low overall satisfaction. Sourouni et al., using the same survey framework, also highlighted that mental health symptoms are common yet frequently overlooked [23]. Consistent with this, our cohort rated satisfaction with mental health support among the lowest of all domains, despite depression being a strong determinant of overall dissatisfaction. Together, these findings emphasize that symptoms often considered "cosmetic" by clinicians may in fact represent core burdens for patients, and that psychosocial aspects of PCOS care remain inadequately addressed.

Our results have important clinical implications. They highlight a misalignment between clinical priorities, which frequently focus on metabolic risk, and the symptoms patients report as most distressing. The 2023 international PCOS guideline underscores the need for multidisciplinary care, integrating metabolic, reproductive, dermatologic, and psychological expertise [1]. Yet, our data suggest that implementation of these recommendations remains incomplete, particularly with regard to psychosocial and aesthetic concerns. Given the high prevalence and impact of hirsutism, alopecia, and body image dissatisfaction, PCOS care should incorporate psychodermatologic services, mental health screening, and body image counselling into routine management.

This study has several strengths, including its large sample size, the development of a questionnaire aligned with guideline-recommended PCOS domains, and the simultaneous assessment of both symptom frequency and patient-centered outcomes. The REDCap-based online design minimized recall and response bias. Nonetheless, certain limitations should be acknowledged. Reliance on self-reported diagnoses of PCOS and metabolic conditions may have introduced misclassification, and the cross-sectional design precludes causal inference. Satisfaction scores served as a proxy for perceived burden but may also reflect broader health system factors, such as access to care, provider communication, and cultural expectations. Selection bias is possible, as participants may differ from the broader PCOS population in symptom profile or satisfaction levels. Finally, validated instruments for assessing quality of life were not employed, and varying response rates across items may have reduced statistical power.

## Clinical implications

Our findings highlight a fundamental misalignment between what clinicians prioritize and what patients experience as burdensome, particularly in the psychosocial and aesthetic domains. The 2023 international PCOS guideline emphasizes the need for multidisciplinary care, including dermatologic, metabolic, reproductive, and psychological expertise [1]. However, our results suggest that implementation of these recommendations in routine practice remains limited, especially with respect to integrating dermatologic and psychosocial support. Addressing these gaps will be essential to ensure that guideline-based care reflects the concerns most relevant to patients.

Given the high prevalence and impact of symptoms such as hirsutism, alopecia, and body image concerns, PCOS care should integrate psychodermatologic services, mental health screening, and body image counselling into routine management.

## Strengths and limitations

This study’s strengths include its large sample size, the use of a questionnaire designed around the full spectrum of guideline-recommended PCOS domains, and the inclusion of both symptom frequency and patient-centered outcomes. The REDCap-based design minimizes recall and response bias.

Several limitations should be acknowledged. First, reliance on self-reported diagnoses of PCOS and metabolic conditions may have introduced misclassification. Second, the cross-sectional design limits causal inference. Third, although satisfaction scores provide a proxy for perceived burden, they may be influenced by unmeasured contextual factors—such as access to care, wait times, provider communication, and cultural expectations—rather than symptom severity alone. Dissatisfaction may also reflect broader health system challenges in addition to individual disease burden. In addition, selection bias is possible, as women who elected to participate may differ in age, symptom profile, and particularly satisfaction levels compared with the broader PCOS population. Finally, the survey did not employ validated instruments for assessing quality of life, and variable response rates across items may have reduced statistical power.

## Supplementary Information

Below is the link to the electronic supplementary material.

Supplementary file1 (PDF 354 KB)

Supplementary file2 (PDF 89 KB)

Supplementary file3 (PDF 905 KB)

## Data Availability

No datasets were generated or analysed during the current study.
