# Supplementary material for: Obesity and dysglycemia independently predict symptom burden but not satisfaction with care in polycystic ovary syndrome: a cross-sectional study
Source: Arch Gynecol Obstet. 2025 Oct 17;312(6):2241–50. doi: 10.1007/s00404-025-08219-9 (PMC12705805; doi:10.1007/s00404-025-08219-9)
Supplement: Supplementary file 1 — Supplementary file1 (PDF 354 KB) [file 404_2025_8219_MOESM1_ESM.pdf]

Supplementary Figures:

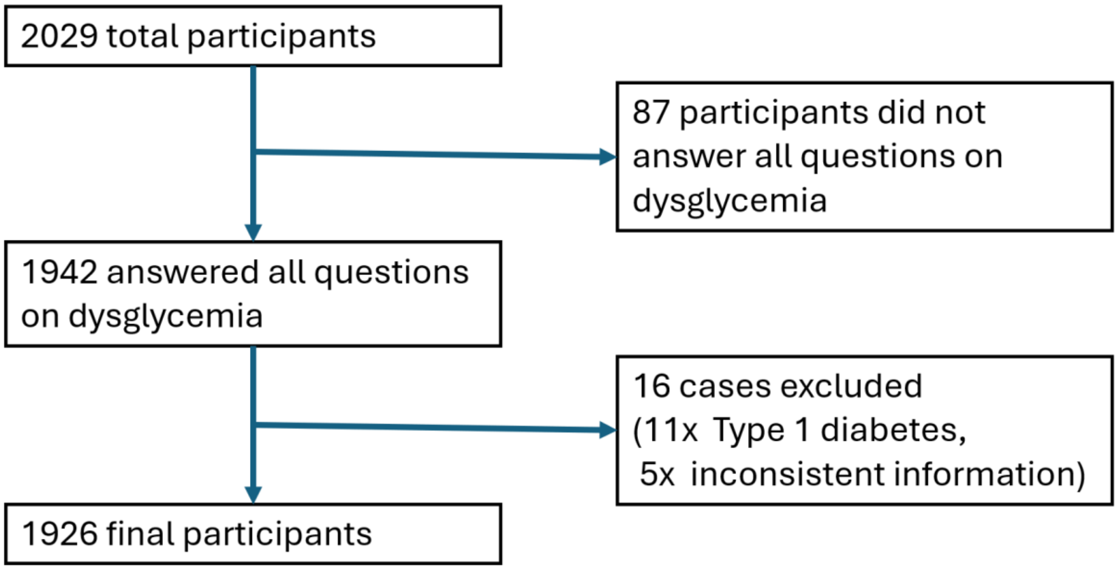

Suppl. Figure S1: Flowchart of Participant Inclusion

Flow diagram illustrating the selection process of study participants. Of the 2,029 total participants, 87 were excluded for not answering all dysglycemia-related questions, leaving 1,942 participants. An additional 16 cases were excluded due to a diagnosis of Type 1 diabetes (n = 11) or inconsistent information (n = 5), resulting in a final study population of 1,926 participants

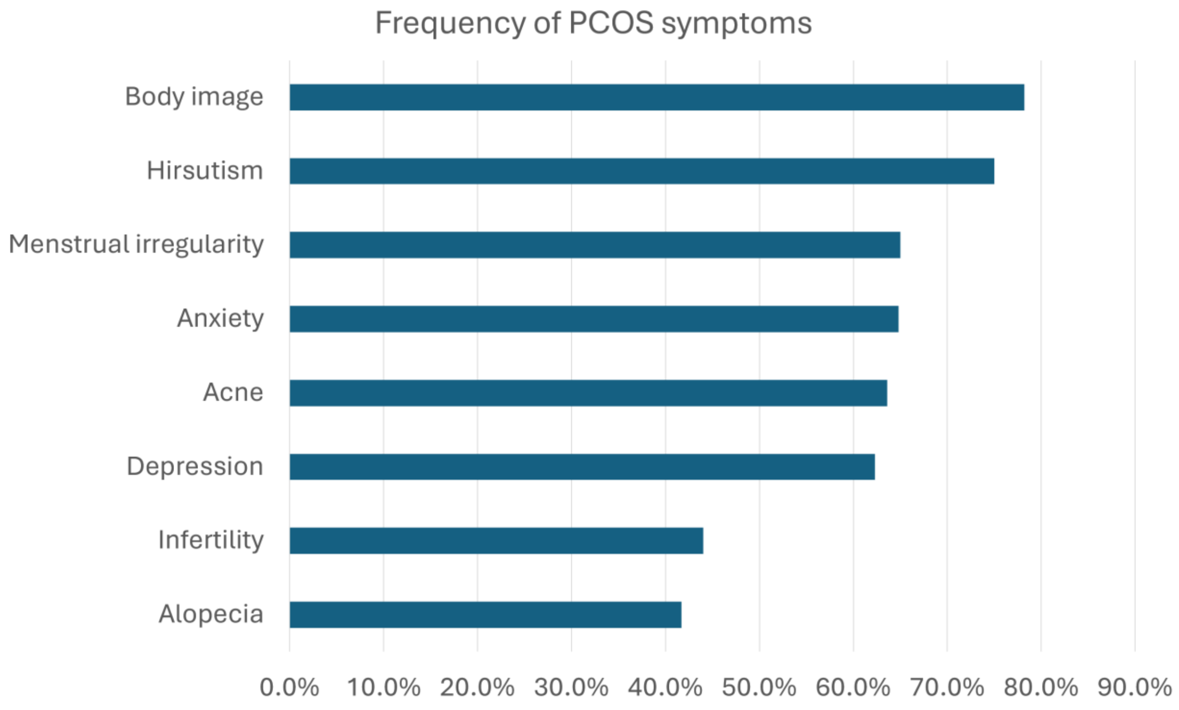

Suppl. Figure S2: Prevalence of common PCOS symptoms

Bar chart illustrating the frequency of self-reported symptoms among women with polycystic ovary syndrome (PCOS). The most frequently reported symptoms were body image concerns (79.1%), hirsutism (75.3%), menstrual irregularity (64.8%), anxiety (64.4%), acne (63.9%), and depression (63.0%). Infertility (43.3%) and alopecia (41.9%) were less frequently reported

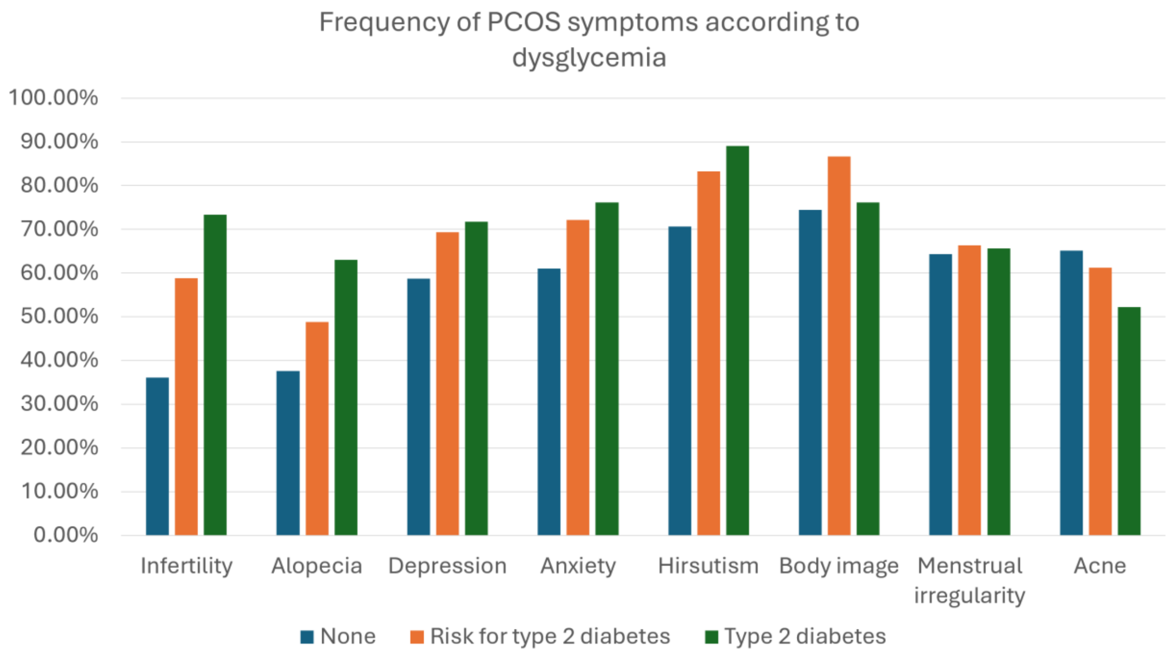

Suppl. Figure S3: Prevalence of PCOS Symptoms by Dysglycemia Status

Bar chart showing the frequency of self-reported symptoms among women with polycystic ovary syndrome (PCOS) according to glycemic status: no dysglycemia, at risk for type 2 diabetes (elevated fasting glucose level and/or gestational diabetes) and type 2 diabetes. Across most symptoms, prevalence was highest in participants with type 2 diabetes, followed by those at risk for type 2 diabetes, and lowest in those without dysglycemia. Notably, hirsutism and anxiety were most frequent in participants with type 2 diabetes, while body image concerns were most prevalent among those at risk for type 2 diabetes.

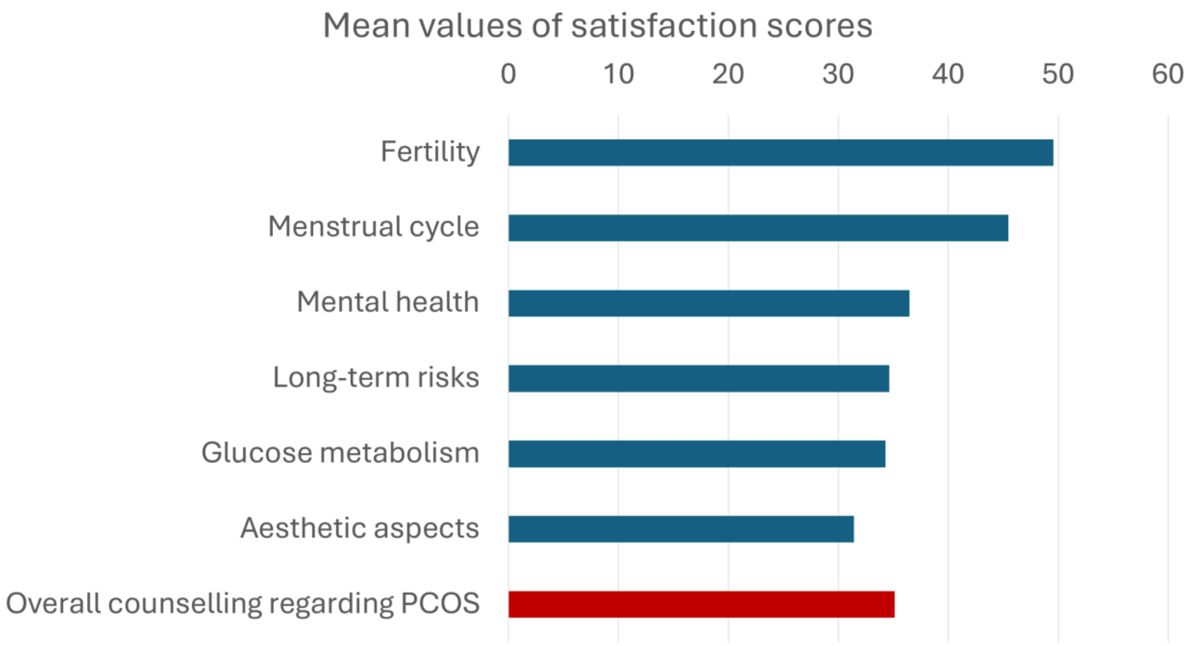

Suppl. Figure S4: Mean satisfaction scores with Counselling on PCOS-related topics

Bar chart showing the mean satisfaction scores (0–100 scale) reported by women with polycystic ovary syndrome (PCOS) for counselling received on various health aspects. Highest satisfaction was reported for fertility-related counselling (mean score ~50), followed by menstrual cycle, mental health, long-term risks, glucose metabolism, and aesthetic aspects. Overall counselling satisfaction regarding PCOS was lower than fertility-specific counselling, highlighting potential gaps in comprehensive patient education.
