# Supplementary material for: Obesity and dysglycemia independently predict symptom burden but not satisfaction with care in polycystic ovary syndrome: a cross-sectional study
Source: Arch Gynecol Obstet. 2025 Oct 17;312(6):2241–50. doi: 10.1007/s00404-025-08219-9 (PMC12705805; doi:10.1007/s00404-025-08219-9)
Supplement: Supplementary file 2 — Supplementary file2 (PDF 89 KB) [file 404_2025_8219_MOESM2_ESM.pdf]

Supplementary Tables:

Suppl. Table S1: Association of PCOS symptoms and dysglycemia

Main PCOS symptoms		None	Risk for type 2 diabetes[a]	Type 2 diabetes	P-value*
Infertility	No	804 (63.9%)	241 (41.2%)	12 (26.7%)	**<0.001**
	Yes	455 (36.1%)	344 (58.8%)	33 (73.3%)	
Alopecia	No	801 (62.4%)	305 (51.2%)	17 (37.0%)	**<0.001**
	Yes	483 (37.6%)	291 (48.8%)	29 (63.0%)	
Depression	No	530 (41.3%)	183 (30.7%)	13 (28.3%)	**<0.001**
	Yes	754 (58.7%)	413 (69.3%)	33 (71.7%)	
Anxeity	No	501 (39.0%)	166 (27.9%)	11 (23.9%)	**<0.001**
	Yes	783 (61.0%)	430 (72.1%)	35 (76.1%)	
Hirsutism	No	377 (29.4%)	100 (16.8%)	5 (10.9%)	**<0.001**
	Yes	907 (70.6%)	496 (83.2%)	41 (89.1%)	
Body image	No	329 (25.6%)	80 (13.4%)	11 (23.9%)	**<0.001**
	Yes	955 (74.4%)	516 (86.6%)	35 (76.1%)	
Menstrual irregularity	No	319 (35.7%)	152 (33.7%)	11 (34.4%)	0.762
	Yes	574 (64.3%)	299 (66.3%)	21 (65.6%)	
Acne	No	448 (34.9%)	231 (38.8%)	22 (47.8%)	0.071
	Yes	836 (65.1%)	365 (61.2%)	24 (52.2%)	

[a]Risk for type 2 diabetes: elevated fasting glucose level or history of gestational diabetes

**Bold indicates statistical significance*

Suppl. Table S2: Correlation of individual satisfaction scores

Satisfaction with counselling regarding		Overall	Mental health	Menstrual cycle	Repro-duction	Aesthetic aspects	Metabo-lism
**Long-term risks**	Pearson corr.	0.684	0.585	0.541	0.498	0.472	0.504
	P-value	<0.001	<0.001	<0.001	<0.001	<0.001	<0.001
	N	1786	1819	1819	1819	1819	608
**Glucose metabolism**	Pearson corr.	0.661	0.542	0.563	0.559	0.537	
	P-value	<0.001	<0.001	<0.001	<0.001	<0.001	
	N	601	620	635	630	642	
**Aesthetic aspects**	Pearson corr.	0.599	0.521	0.555	0.457		
	P-value	<0.001	<0.001	<0.001	<0.001		
	N	1786	1856	1913	1889		
**Fertility**	Pearson corr.	0.629	0.543	0.633			
	P-value	<0.001	<0.001	<0.001			
	N	1786	1856	1889			
**Menstrual cycle**	Pearson corr.	0.674	0.548				
	P-value	<0.001	<0.001				
	N	1786	1856				
**Mental health**	Pearson corr.	0.634					
	P-value	<0.001					
	N	1786					

Suppl. Table S3: Linear regression of the overall satisfaction score by individual satisfaction scores

Satisfaction with counselling regarding	Coefficient B	95% CI	P-value*
Long-term risks	0.261	0.203-0.320	**<0.001**
Glucose metabolism	0.187	0.131-0.244	**<0.001**
Aesthetic aspects	0.163	0.100-0.226	**<0.001**
Fertility	0.162	0.107-0.216	**<0.001**
Menstrual cycle	0.135	0.072-0.197	**<0.001**
Mental health	0.050	-0.007-0.106	0.084

**Bold indicates statistical significance*

Suppl. Table S4: Correlation of satisfaction scores with BMI classes

Satisfaction with counselling regarding	Spearman Rho	P-value*	N
Mental health	-0.113	**<0.001**	1855
Fertility	-0.112	**<0.001**	1888
Aesthetic aspects	-0.109	**<0.001**	1925
Glucose metabolism	-0.091	**0.022**	642
Menstrual cycle	-0.071	**0.002**	1912
Long-term risks	-0.015	0.517	1818
**Overall**	-0.063	**0.007**	1785

**Bold indicates statistical significance*
